# Cognitive and Neurocognitive Effects From the Unique Bilingual Experiences of Interpreters

**DOI:** 10.3389/fpsyg.2020.548755

**Published:** 2020-09-29

**Authors:** Aline Ferreira, John W. Schwieter, Julia Festman

**Affiliations:** ^1^Bilingualism, Translation, and Cognition Laboratory, University of California, Santa Barbara, Santa Barbara, CA, United States; ^2^Language Acquisition, Multilingualism, and Cognition Laboratory, Wilfrid Laurier University, Waterloo, ON, Canada; ^3^Multilingualism Research Team, Institute for Research and Development (IFE), University College of Teacher Education, Tyrol, Austria

**Keywords:** bilingualism, bilingual experience, cognitive benefits of bilingualism, working memory, simultaneous interpreting

## Abstract

For bilinguals, research suggests that both languages are constantly active and competing in the mind, even when only using one. However, this body of work has reported inconclusive results on the long-term effects of the constant parallel activation and use of more than one language on the brain. This has mostly been due to inconsistent comparisons between groups of bilinguals and monolinguals. Not all bilingualisms are the same. The investigation of the use of more than one language over a lifetime offers the opportunity to better understand the consequences of bilingualism on the brain. However, few studies have specifically looked at the long-standing effects of being an interpreter. In this paper, we review theories from the field of Translation and Interpreting Studies and provide a critical review of work that offers insight on the cognitive and neurocognitive effects that seem to arise from the unique, highly-cognitive-demanding practices experienced by interpreters.

## Introduction

Translation and interpreting are two special subtypes of bilingual communication. While translation is conveyed through written language – from one text into another – interpreting involves the immediate verbal communication from one language to another. Not only do these two differ in what they accomplish, but each of them also has its own subfields. Literary, technical, scientific, financial, legal, and medical translation, for instance, all have unique, domain-specific differences with specialized vocabulary linked to each. In other words, the requirements for professional translators go above and beyond simply being highly competent in two languages. The same can be said for interpreting: simultaneous, consecutive, and sight interpreting are offered in different settings (medical, judicial, business, etc.), and these three modes also require specific knowledge and training because they each entail different skill sets.

There is one common characteristic to all of these: bilingualism. Research has shown that for bilinguals, both languages are active (to different degrees) in the mind, even when only using one (Marian and Spivey, 2003). However, this body of work has reported inconclusive results with respect to how this constant parallel activation and use of more than one language affects cognition and the brain. This mostly has been due to inconsistent comparisons between groups of bilinguals and monolinguals and, to some degree, the (unconscious) belief that all bilingualisms are the same. Different bilingual cohorts including simultaneous interpreters (SIs), consecutive interpreters, among others, possess different skill sets that are unique to the needs of their professions. The investigation of their language use over a lifetime offers the opportunity to better understand the consequences of bilingualism on the brain. For instance, simultaneous interpreting (SI) involves the management of a wide array of cognitively-demanding tasks at rapid speed, including comprehension, simultaneous speech perception and production, and attention switching. Some research has shown that already shortly after training in these skills, there are indications of anatomical and functional changes in the brain (van de Putte et al., 2018).

However, few studies have specifically looked at the long-standing effects of being an interpreter. In this paper, we first provide a review of theories from Translation and Interpreting Studies followed by a discussion on how experience with interpreting may have important neurocognitive effects that are shaped by the unique practices of interpreters, which other bilinguals and monolinguals do not have.

## Bilingualism and Linguistic Competence of SIs

Grosjean (1994) defines a bilingual as any person who uses two languages or dialects on a regular basis (see also Calvo et al., 2016). Calvo et al. argue that this broad definition allows bilinguals to be “classified in terms of age of L2 acquisition (early vs. late bilinguals), simultaneity of L2 acquisition (simultaneous vs. sequential bilinguals), L2 proficiency (from incipient to low-, mid-, and high-proficiency bilinguals), and frequency of L2 use (active vs. latent bilinguals), among other variables” (para. 6). Harris (1977) argues that all bilinguals possess three competences: competence in the L_*i*_, competence in the L_*j*_, and a third competence, that of translating from one language into another. That is what is called natural translation, which is defined by Harris as a natural skill that, like all skills, is capable of improvement under guidance.

Valdés and Angelelli (2003, p. 58) describe interpreters as a group of “language-using individuals, who, as the locus of language contact, alternately use two languages at the same time and in the same place to broker communication” between individuals who do not speak the target language. Such bilinguals are unique because they do not choose one or the other language depending on a set of factors: they work in a variety of settings and “use their two languages to convey the spoken discourse of individuals who speak one of their languages to individuals who speak their other language” (p. 59). Few studies have addressed what constitutes the “perfect grasp” – a reference to Henderson’s (1982) reading on what an interpreter must master in terms of the languages themselves in order to interpret. We are left with a broad set of guidelines put forth by professional organizations and based on research on interpreting training.

The American Translation Association (ATA, 2014) guide suggests that interpreters need to take a multistep approach to obtain a specific certification, which includes a language test for languages in which there are no interpreting skills available. Those tests are “a reliable way to assess foundational language skills necessary for interpreting. However, these oral language proficiency tests do not evaluate interpreting skills” (p. 8). The guide also argues that competencies in intercultural communication and language skills are closely related, but there are few tests available for measuring intercultural communication skills. However, there are more tests accessible that evaluate oral and written skills, such as the Interagency Language Roundtable, which the ATA states is:

A collaborative effort of US federal government agencies, academia and language specialists, has developed a 6-point skill level scale to evaluate language proficiency. The American Council on the Teaching of Foreign Languages (ACTFL) adapted this scale for use in academic settings and the two organizations currently work together to ensure that the two systems are complementary. The ACTFL exams, along with the Defense Language Proficiency Tests (DLPT) developed by the US Department of Defense and the European Common Framework for Languages testing, are an effective way to determine proficiency in numerous languages (p. 11).

The ATA further presents a description of translator competencies which can apply to interpreters. The first is *language proficiency* (reading and writing), which refers to the need for high-level reading proficiency in the source language and writing proficiency in the target language. The second competency is *translation skills proficiency*, related to the skills involved in transferring a message from one language to another while maintaining the same meaning in the target language. *Cultural proficiency* is defined as the ability to participate in social situations while understanding what is being communicated and employing appropriate pragmatics to convey a message. Finally, *domain expertise* refers to the advanced knowledge of the subject matter of the source text.

Napier et al. (2005) analyzed Australian Sign Language and perceptions of bilingual status among interpreters of English. The researchers compared these perceptions to their preferences for direct or inverse interpreting. The study questioned just “how bilingual” an interpreter must be in order to interpret effectively. Interestingly, results showed that many interpreters’ perception and preferred language direction contravened their established practices (i.e., they preferred to interpret into the less-dominant language but typically interpreted into their first language). Napier et al. also present a review on directionality and how an interpreter’s two languages are viewed in the professional world. Interpreters have their own native language (Language A), a language other than the native but of which they have a perfect command (Language B), and a language of which they have full understanding and from which they work (Language C). However, this type of description lacks scientific analysis and seems to be largely subjective. Future work will need to offer empirical analyses that support or refute these claims. In the next section, we will discuss some of the theories – specifically from a processing perspective – that describe these competences and other phenomena in interpreting.

## Theories From Interpreting Studies

In interpreting studies, research has focused mostly on teaching of and training in interpreting, although there has been an increase in empirical-based work since the 2000s. Gerver (1975) notes that interpreting is a “very complex behavior…(that) can shed on more general aspects of human attention, memory, and linguistic behavior” (p. 119). Gerver (1976) proposed a model based on information processing to describe the mental operation of SI. This was the first model that considered both short- and long-term memory in SI. Gerver (1976) examined the role of input procedures, working memory (WM), decoding and encoding, and output procedures in SI. The study focused on several short-term stores for the various stages of text processing. The findings suggest that an input buffer stores a segment of the source while simultaneously processing the next segment. At this stage, processing is purely linguistic, ignoring any other type of information. Next, the processed material proceeds to an output buffer. Important to Gerver’s model is that it is based on two buffers, one for each language, and that processing occurs via multiple channels.

Moser’s (1978) model describes the crucial role of WM in interpreting. She explains that the message in the source language is first received by the auditory receptor system. It then becomes available for feature detection to determine whether an acoustic feature is presented, and the information is stored in the perceptual auditory storage. Using the phonological rules of the source language, a primary recognition process takes place in order to organize the acoustic features into a synthesized percept. The latter is stored in synthesized auditory memory. Then, a second process starts: the secondary recognition process will transform the sequence of synthesized syllables into words, in which “Syntactic and semantic cues are necessary for word recognition to occur; their possible nature will be described together with the explanation given for concepts in long-term memory” (p. 354). Moser uses generated abstract memory to explain short-term memory. Generated abstract memory stores processed chunks of text and performs a recoding task in co-operation with a conceptual base. The conceptual system is strongly linked to long-term memory through those operations, as long-term memory is responsible for storing all concepts, syntax and grammar rules, and lexicon.

According to Gile’s (1995) Effort Models, operational components of interpreting are pooled together into four types of effort: listening and analysis, short-term memory, production, and coordination. Listening and analysis effort consists of “all comprehension-oriented operations, from the analysis of the sound waves carrying the source language speech which reaches the interpreter’s ears, through the identification of words, to the final decision about the meaning of the utterance” (p. 162). Short-term memory effort deals with memory operations from the moment in which a segment is heard to the moment when it is reformulated in the target speech or alternatively disappears from memory. Production effort refers to speech output in simultaneous production in the first stage of consecutive interpreting. Finally, coordination effort is required to harmonize the other three efforts. The term *effort* is used to highlight the non-automatic nature of those components. Each point in time, the efforts will require different processing capacities that should be sufficient to complete the task. This model was proposed to describe the difficulties of an interpreting task and the selection of appropriate strategies and tactics.

In a recent paper by Dong and Li (2019), the researchers review empirical studies that have investigated the demands placed on language control (i.e., ensuring that one language does not interfere with the other) and processing control (i.e., multiple tasks performed under time pressure) during interpreting. Their review culminates in a proposal for an attentional control model for interpreting that accounts for both language and processing control. According to the authors, “since successful performance in interpreting requires processing control, processing control may get strengthened by continued practice in interpreting” (p. 8). Also, processing control is achieved “mainly through coordination, WM, and language processing,” in which case training in interpreting would enhance “coordination ability, WM capacity, and language processing efficiency” (p. 9). The general attention control model is a theoretical explanation of language control and cognitive control that are supervised by attentional control (also referred to as a supervisory attentional system, Norman and Shallice, 1986). The attentional control is essential to normal functioning in everyday activities. The proposal for processing control is assumed to be accounted for the multi-tasking nature of interpreting, attempting to investigate the frequency and effects of switching between listening in one language and conveying the information in another language.

Although the models discussed above have helped us to better understand language processing in interpreting, they call for more empirical research in order to identify details of processing that could potentially explain their proposals. Furthermore, not one has explicitly engaged in a conversation on the role of bilingualism.

## The Unique Bilingual Experiences of Interpreters

Our daily activities have profound consequences for cognition. In the case of SIs, such activities include unique, professional use of their languages, namely utilizing one language for auditory perception while using the other language for verbal production. SI involves complex parallel processes that allow for the perception, storage, recall, and transformation of auditory input. SIs must also anticipate upcoming utterances in the input (Dong and Xie, 2014) and incorporate form-level and meaning-based processing during interlingual reformulation (Paradis, 1994). Henrard and van Daele (2017) describe SI as being:

Highly demanding in terms of executive control, requiring a large number of cognitive functions and processes to be activated simultaneously under heavy time pressure (Christoffels et al., 2006; Köpke and Nespoulous, 2006). This activity requires to continuously receive new information while simultaneously understanding speech, storing it in memory, and producing a translation of an earlier portion of speech (Gerver, 1976; Lambert, 1992; Moser-Mercer, 2000). Indeed, the simultaneous interpreter must listen to and understand speech in one language, holding it in memory until it is re-encoded to be produced in another language. At the same time, the interpreter utters the translation of a portion of speech encoded earlier (para. 6).

In SI, time pressure increases the level of cognitive demand and, thus, the executive processes required for the activity. Translators, contrastively, are not affected by the flow of auditory input in the source language and do not need to process information under time pressure. This in turn causes a difference in the processing speed and the speed at which information received has to be updated and increases the likelihood of cross-language interference more than in ordinary bilinguals (Gile, 2009). Many studies have shown that any activity with time pressure leads individuals to adopt two strategies: acceleration of information processing and filtering of information (Edland and Svenson, 1993; Maule et al., 2000). Under time pressure, it is difficult for interpreters to process all the information, and as such, they intentionally ignore irrelevant information. Using conference interpreting as a point of departure, Obler (2012) states that “the extreme language demands of (interpreters’) task provide us, as exceptional groups and exceptional performance often do, a useful tool to determine the ways the brain engages cognition to process and produce language” (p. 177).

Time pressure seems to play a different role in consecutive interpreting (CI) than in SI. In fact, some consecutive interpreters may “stay several seconds behind the speaker” and others may wait for the “speaker to stop speaking in order…to deliver the message” (Russell, 2005, p. 136). This is strikingly different than SI given that consecutive interpreters receive speech input that are at least a couple of sentences at a time. Thus, whereas time pressure plays a significant role in the cognitive effort put forth in SI, working memory is key in CI. Liang et al. (2017) compared the effect of sentences complexity (as operationalized by dependency distance, i.e., “linear distance between two syntactically related words in a sentence,” p. 1) on the cognitive constraints involved in SI and CI. The results showed that CI entails heavier cognitive demands than SI because it “requires a non-simultaneous, but sequential alternation between listening and speaking” (p. 7) (see also Christoffels and de Groot, 2005). Liang et al. proposed a revised framework to Gile’s Effort Model (Gile, 2016) that reflects the correlation between sentence formation and demands from time constraints.

Work by Macnamara (2012) has significantly informed our knowledge of the foundational capabilities that are needed to perform the cognitive functions involved in SI. In, the author examined the aptitude of several variables among SI trainees including “spoken and signed language interpreter processing; second language acquisition; and cognition, specifically memory, intelligence, information and language processing, decision-making, problem solving, multitasking, skill acquisition, expertise, and human performance” (p. 9). The theoretical frameworks of interpreting aptitudes and of second language learning aptitudes have served as a springboard for other studies. For instance, Macnamara and Conway (2016) examined whether the amount of training in SI, the trainees’ cognitive abilities, and their initial SI performance could predict the final SI performance at the end of 2 years of training. Although all of these factors positively predicted final SI performance, WM was a significantly stronger predictor with more consistent results than the other factors. Similar results were reported by Macnamara et al. (2011) who found that personality characteristics such as “risk-taking orientation and emotion-cognition integration style, and intrinsic motivation to engage in complex cognitive tasks” (p. 107) also predict SI performance.

In Hiltunen et al. (2016), WM and executive control of SIs, consecutive interpreters, foreign language teachers, and non-interpreter bilinguals were compared. The results from a free-recall task and a cocktail-party dichotic listening task demonstrated expertise-dependent differences between all four groups that can be explained by their conditions at work. For SIs, there are demanding linguistic tasks in which attention is continuously divided up between listening to the source text, formulating and uttering the output, and monitoring the equivalence of both. For consecutive interpreters, there are high demands for resisting external distractions at work. The general implication from Hiltunen et al.’s study is that these differences seem to be a reflection of the distinct experiences in each of their professional fields.

Timarová et al. (2014) studied WM executive control among SIs on a battery of four central executive tasks exploring inhibition (though a flanker task and anti-saccade task), updating (using a 2-back task), and shifting (using a number-letter task) during three simultaneous interpretations. The results suggested that although some WM functions seem to be related to interpreting experience, others such as automatic response inhibition, updating, and attention switching are not. Overall, the authors argue that attentional control is an important component of the SI process. Timarová et al. (2015) further elaborate this argument by testing the same cohort of interpreters on WM capacity (letter span, Corsi task, complex span) and on several measures of interpreting performance (lexical, semantic and syntactic processing, temporal delay, vocabulary richness, and dealing with speed). The findings showed a dissociation between verbal and spatial memory and a negative correlation between age, WM measures, and general cognitive ability. The implications to be drawn from these two studies is that “processes tapping the storage component of WM do not seem to play a crucial role in professionals with a higher degree of skill acquisition” (p. 123) (see also Köpke and Nespoulous, 2006; Köpke and Signorelli, 2012). These highly-cognitive-demanding experiences in two (or more) languages are particularly interesting in their long-term impact on cognition (in contrast to more short-term effects of training), since experimental studies including conditions of high cognitive demands revealed cognitive superior performance (Costa et al., 2009; Diamond, 2014). In the next section we will review the potential cognitive and neural effects, specifically among SI professionals, both short-term and long-term, that may arise from the parallel employment of language and processing control.

## Cognitive and Neurocognitive Effects of Interpreting

### A Cognitive Advantage for Interpreters?

García (2014) put forward the hypothesis of an “interpreter advantage,” which suggests that the daily training and long-term use of such a unique combination of cognitive and linguistic demands a professional interpreter is faced with should result in enhancement of linguistic and cognitive skills, but only those closely related to these very specific, profession-related demands. Due to a special, dual-language condition that is present right from the start of interpretation training, García (2014) concluded that aspects of the interpreter advantage develop shortly after the onset of formal intense training, rather than after several years of experience in the profession (see Köpke and Nespoulous, 2006; Elmer et al., 2010), as for trainees it is necessary to execute interpreting tasks properly from the very beginning. These training-induced changes (early changes as described in section “Effects of Training in Interpreting”) might leave their trace and substantiate in the professional, highly experienced interpreter’s brain (see section “Effects of Training in Interpreting”). Of course, changes in neurocognitive systems are the consequence of sustained use and long-term professional coping with the specific professional demands. Moreover, behavioral advantages were found to correlate with time on task, i.e., the hours of practice (Elmer et al., 2014), and with years of professional experience (Santilli et al., 2019). García (2014) therefore speculates that these rather task-specific skills may result in more efficient abilities in the linguistic and cognitive domain and be observable even in non-interpreting tasks, but only in restricted linguistic and cognitive subdomains (see García et al., 2019, for review).

Since SI is first of all based on the perception of key information in the incoming sound stream and the comprehension of the constantly unfolding speech, SI may have trained the specific skills taxed in professional settings. Thus, only some bilingual verbal skills are behaviorally enhanced. For the auditory domain, Elmer et al. (2014) showed enhanced auditory perception (verbal and non-verbal sounds) for expert SI compared to professional musicians and non-expert controls. This might point at superior abilities to extract and recognize relevant information from complex auditory input.

Other advantages have been found on the more conceptual level, namely for sentence comprehension (Bajo et al., 2000), and in tasks in which expert interpreters were tested in understanding (Yudes et al., 2013) and recalling (Dillinger, 1990) longer pieces of discourse. Superior performance has been observed specifically in detecting semantic errors (Fabbro et al., 1991; Yudes et al., 2013). This ability may stem from their professional need to properly comprehend input, which apparently resulted in greater sensitivity to semantic rather than syntactic features and in general in a superior capacity to understand unfolding texts.

Storage of information and keeping it transiently active is crucial for expert SI due to the delay between input and output and the continuous new information. Studies have shown better short term and working memory in terms of larger memory spans (reading span, word span, and speaking span) when concurrent cognitive operations (e.g., mathematical calculations, speaking, reading aloud) were included (Christoffels et al., 2006; Yudes et al., 2011, 2013; Signorelli et al., 2012; Babcock and Vallesi, 2017). Signorelli et al. (2012) compared younger and older interpreters and non-interpreters on reading span, non-word repetition, and order- and category-cued recall abilities. The results showed that interpreters outperformed non-interpreters on reading span and non-word repetition but not on cued recall. The authors interpret this as evidence that interpreters are better at manipulating information in WM and processing sub-lexical phonological representations but not at short-term retention of words. This ability to store new information and to process it in parallel is considered a benefit from the systematic entrenchment of SIs’ profession.

Babcock and Vallesi (2017) recently asked an important question as depicted in the title of their article, “Are simultaneous interpreters expert bilinguals, unique bilinguals, or both?” The results of the study did not reveal advantages in conflict resolution or switching cost for interpreters where previous advantages among non-interpreter bilinguals have been found. However, the interpreters showed interpretation-specific advantages along with larger verbal and spatial memory spans compared to non-interpreter bilinguals. The authors conclude that “interpreters do not continue to garner benefits from bilingualism, but they do appear to possess benefits specific to their experience with simultaneous interpreting” (p. 403).

In contrast to trainees, expert SI show this memory advantage more generally: for shapes (Babcock and Vallesi, 2017), digits (Bajo et al., 2000; Stavrakaki et al., 2012; but see Köpke and Nespoulous, 2006; Santilli et al., 2019, for contradicting findings), letters (Babcock and Vallesi, 2017), non-words (Stavrakaki et al., 2012), words in L1 and L2 (Christoffels et al., 2006), and for updating (Henrard and van Daele, 2017). The investigation of recall abilities in professional SI has shown that only if the task involves articulatory suppression (what is similar to the professional task of SI), expert SI have a larger recall capacity than other multilinguals (Bajo et al., 2000; Köpke and Nespoulous, 2006; Yudes et al., 2012). If rehearsal is not impeded, they perform similarly to control groups (e.g., Hiltunen et al., 2016).

Interlingual reformulation, production, and monitoring of the translation are yet another set of specific skills trained in expert SI. Probably related to the enhanced training of retrieval of highly specific lexical items when time for lexical search is scarce, comprising intra- and inter-linguistic lexical search, professional SI showed superior performance when tested for word knowledge/wider vocabulary (Christoffels et al., 2006), phonological and semantic verbal fluency (Santilli et al., 2019), word translation (Santilli et al., 2019), and for manipulation of non-words. Interestingly, expert SIs were better at recognizing (Bajo et al., 2000), repeating (Signorelli et al., 2012), and recalling non-words (Stavrakaki et al., 2012). As mentioned earlier, SIs need to be prepared for the unexpected and anticipate upcoming utterances in a constantly unfolding speech under time pressure. Hence this advantage in efficient within- and cross-language processing and managing unfamiliar information can be explained, such as the translation of highly infrequent words.

Since expert SI do not need to switch between their languages in one modality but rather use one in the auditory modality and the other in the verbal production modality, there seems not to be any need for alternating inhibition. Both languages need to be active in parallel, but for the processing in one specific modality. In general, inhibition is apparently not a skill trained specifically in SI (Ibáñez et al., 2010, but see Henrard and van Daele, 2017, for different types of inhibition, in particular resistance of proactive inhibition), since tasks assessing interference control did not show an interpreter advantage, e.g., the Stroop (Köpke and Nespoulous, 2006) or the Simon task (Yudes et al., 2011).

When having to manage concurrent tasks such as the joint input and output demands, great mental coordination skills are necessary. Superior processing of concurrent WM content is associated with experience in interpreting (Padilla et al., 2005). Studies have also shown that expert SI outperform control groups in dual task performance when having to divide attention and allocate mental resources to two different, unrelated processes that have to be executed in parallel. One such laboratory test comprises the presentation of auditory and visual stimuli (Morales et al., 2015; Strobach et al., 2015; Becker et al., 2016). Monitoring the processing of multiple tasks is furthermore crucial for expert SI. Morales et al. (2015) showed superior performance in monitoring and updating for SI (n-back task in an easy and a dual-task condition).

Shifting focus from one task to another is yet another skill in which expert SIs are trained by profession and which yielded observable differences to control groups on tasks assessing mental-set shifting (e.g., between discriminating color and shape) and switching (Henrard and van Daele, 2017), showing also significantly lower mixing costs than control groups (Becker et al., 2016; Babcock and Vallesi, 2017). More generally, Henrard and van Daele (2017) stressed the speed of processing as one remarkable characteristic of expert SI processing hinting at advantages linked to years of experience. Similarly, Christoffels et al. (2006) revealed faster performance on a naming task.

### Effects of Training in Interpreting

We have established that the language experiences of SIs are unique from other bilinguals, but do these experiences offer additional cognitive and/or neurocognitive benefits above and beyond typical bilingualism? If so, is this because of their intensive training to become an interpreter, their years of experience being an interpreter, or both? Here we briefly describe some studies that have investigated the effects that training in interpreting has on cognition and particularly on WM and shifting.

Just as SI is an “extreme form of bilingualism” (van de Putte et al., 2018, p. 243), the training required to become a professional interpreter is equally intense. In fact, recent studies have shown that cognitive effects associated with experience in interpreting can be observed shortly after beginning their training (Köpke and Nespoulous, 2006). Most of these findings have come from studies employing WM tasks that require systematic parallel processing. Antonova Ünlü and Sağın Şimşek’s (2018) longitudinal study over five semesters of training demonstrated that such training not only improved interpreting skills of trainees but also their central executive and processing capacity for WM. Similarly, Chmiel (2018) reported that interpreter training improves WM capacity, which in turn predicts interpreting performance (c.f., Rosiers et al., 2019).

Notwithstanding, Tzou et al. (2012) compared the WM capacity of interpreting students in their first year of training to that of interpreting students in their second year. While WM span was slightly higher for the students in the second year, this difference was not significant. However, WM was significantly higher for interpreting students with greater L2 proficiency, prompting the researchers to argue that language proficiency may explain differences in interpreting performance and WM and that language processing skills (rather than WM) may be enhanced by formal training in interpreting.

Dong and colleagues have investigated the effects of training in consecutive interpreting that “requires both languages to be constantly active rather than alternately inhibited” (García et al., 2019, p. 8). For instance, Dong and Xie (2014) found that students with more interpreting training outperformed those with little to no interpreting training in the Wisconsin Card Sorting Task, but not in the Flanker task. They argued that language interpreting experience significantly contributes to mental set shifting enhancement in cognitive control but not to inhibition. Other studies using tasks of attentional (Babcock and Vallesi, 2017; Babcock et al., 2017), inhibitory (Köpke and Nespoulous, 2006; van de Putte et al., 2018), and switching (Babcock et al., 2017; van de Putte et al., 2018) skills similarly fail to find an advantage even after 2 years of training. In another study on how training in consecutive interpreting affects WM, Dong et al. (2018) found that as opposed to WM, updating efficiency was enhanced by training. Similarly, Dong and Liu’s (2016) study that compared training in consecutive interpreting to training in written translation showed that training in interpreting produced significant cognitive advantages in switching and updating, whereas training in translation only uncovered marginally significant improvements in updating.

### Neurocognitive Effects Among Trainees, SI, and Highly-Proficient Bi-/Multilinguals

Studies using neuroimaging technology on students enrolled in intensive training programs in interpreting have shown an increase in the volume of gray matter in regions that are involved in semantic processing, learning, motor control, and several domain-general executive functions (Hervais-Adelman et al., 2015). Other research has found that SI trainees develop an increased cortical thickness in temporal, parietal, and dorsal premotor regions that are important to phonetic, lexico-semantic, and executive functions (Hervais-Adelman et al., 2017) and that SI entails “increased activity in frontobasal and perisylvian regions, with maximal recruitment of linguistic and cognitive control hubs (e.g., superior temporal and prefrontal cortices) during parallel processing of input and output (Hervais-Adelman et al., 2014)” (García et al., 2019, p. 2). van de Putte et al.’s (2018) study comparing interpreter trainees and translator trainees before and after their training found a significant increase of the structural connectivity in the frontal-basal ganglia subnetwork, an area typically associated with domain-general and language-specific cognitive control and in the cerebellum and the supplementary motor area, an important region for language control.

Professional interpreters seem to show a specific neuroanatomical and neurofunctional profile of verbal and non-verbal processes. Early neuroanatomical changes related to sustained interpreting practice after years of experience are firstly related to reductions in volume of the bilateral middle anterior cingulate gyrus, the middle anterior insula, the superior middle gyrus, and the pars triangularis, and secondly of changes in gray matter density of these areas as well as of the caudate nucleus, with expert interpreters showing a negative correlation with expertise (measured in terms of hours of practice) (Elmer et al., 2014). Importantly, these areas are key to verbal and non-verbal functions such as WM and phonetic processing, as well as sensory-to-motor coupling. Similarly, changes in gray matter density have been observed as reflections of life-long bilingualism (Abutalebi et al., 2014, 2015).

Contrary to Elmer et al.’s (2014) finding, Becker et al. (2016) reported that the interpreters in their study had left frontal lobes with more gray matter density and volumetric increase in this cognitive control area. The volume was negatively associated with reduced mixing costs in switching tasks, which points at brain-level morphometric changes caused by specific processing demands. Becker et al. (2016) also found changes in functional connectivity for professional interpreters. They reported higher global functional efficiency in the left frontal pole and more functional connectivity to the left inferior and middle temporal gyri during switching and dual-task performance.

Neurofunctional changes have been documented in a study using a semantic decision task based on translation equivalents in different language combinations. While professional interpreters had to judge the semantic correctness of noun-pairs, Elmer et al. (2010) recorded EEG and found enhanced N400 for L1-L1, L1-L2, and L2-L2 noun pair judgments, but not for the commonly trained translation direction L2-L1. The authors suggested training-induced changes in sensitivity to semantic processing in these language conditions compared to the multilingual control group. Moreover, when investigating the functional connectivity in the data of this earlier study, Elmer and Kühnis (2016) found stronger theta-band coupling between the auditory cortex (BA 41/42) and Broca’s area (BA 44/45) when the professional interpreters were performing the task compared to the multilingual controls. Adaptations in relation to professional processing demands were found in terms of a positive correlation between the connectivity pattern and interpreters’ amount of training.

Overall, these studies start to indicate a specific neuroanatomical and neurofunctional profile and stress the experience-related adaptations of the brain as some of the changes were directly related to years of experience in the profession. In the same vein, other researchers have (rightly) advocated that care must be taken when interpreting these results. Calvo et al. (2016) note that “evidence of enhancements induced by interpreting expertise is not entirely robust [and that] studies on possible neuroanatomical changes associated with the bilingual experience have yielded ambiguous results” (para. 9).

## Discussion and Moving Forward

In this paper, we have discussed the nature of the intense and unique bilingual experiences of interpreters and their consequences. We have noted that SI is a highly-complex task that requires several different processes in parallel. Intense training is needed so that SIs can adapt to these cognitive demands. There are robust short-term cognitive effects from training in interpreting but importantly, a clearer picture of how profession-related specific skills leave observable long-term traces in the cognitive and neurocognitive domains starts to emerge. It should be noted that only relatively few empirical studies focusing on brain and cognition of SI have been conducted to date, with relatively small sample sizes, and a variety of tests has been used to evaluate executive control. Henrard and van Daele (2017) therefore conclude that definitive conclusions cannot yet be drawn, especially when comparing participants across dissimilar groups (e.g., with respect to their training, language experiences, age, proficiency, etc.).

One commonly mentioned caveat in bilingualism research (in particular on the bilingual advantage) is the heterogeneity within the samples (age of L2 onset, manner of acquisition, age, proficiency, frequency of language use, SES, migration experience, language switching habits, etc.) (Calvo et al., 2016; see Antoniou, 2019, for a recent review). Kroll and Bialystok (2013) argue that bilingualism must be viewed as a continuous rather than dichotomous variable. Valian (2015) reiterates this view, noting that there is quite a bit of variability among bilinguals with respect to their linguistic, cognitive, and social characteristics, as well as their professional experience and educational background. This dynamic and variable nature of bilingualism makes it difficult to compare groups of bilinguals and draw concrete conclusions.

One may think that because SIs work in the same profession, they may be a more homogenous group. But still, their language acquisition trajectories are different, and often samples are composed of different language pairs, e.g., two Roman languages vs. one Roman and one Semitic language. It is yet unknown what impact language typology has on the interpreter’s advantage (Antoniou and Wright, 2017). Moreover, some of the relevant information on participants’ profile with regard to the type of interpreting and training background is incomplete or unreported. The International Organization for Standardization’s language subcommittee developed the first set of standards for various types of interpreting. For each type of intercepting – conference, media, escort, military, medical, court, etc. – professionals are trained on different techniques relevant to their practice (Bancroft, 2015). This variation in training among participants likely affects the way they perform in experimental tasks. Unfortunately, their training background is usually not disclosed in studies, making it difficult to explain whether their performance is at all related to their background. Not all interpreters are the same. And for the control group, the caveat remains in particular concerning their language use and the frequency of executing tasks similar to interpreting.

One of the remaining questions linked to crucial information on participants’ profile is how to get an objective measure of professional experience. The commonly used indicator in the studies on SI is years of experience. This does not, however, include an estimate of the frequency of actual professional activity, or if SI is a full-time employment or only part-time. The number of hours spent on SI per week or month might diverge greatly between participants; this seems, however, to be a more precise measure of professional experience and should be used in future studies. Other information that often goes unreported but should be included is a description of interpreters’ knowledge of languages other than the two being used in the study. For instance, do they use other languages in their profession, and if so, what is their relative proficiency, what is the directionality of SI for these languages, and how often and in what contexts are these languages engaged? In SI, within-subjects analyses can be conducted under several experimental conditions to ensure maximum control of extraneous participant variables. Researchers can use statistical procedures to reduce cloudy effects on dependent variables, making the data less noisy. Also, although the number of SIs is much less than ordinary bilinguals, every effort should be made to increase sample sizes in studies on SI.

Interpreters possess a unique bilingual life experience, leading to profound consequences for cognition. They are challenged on a daily basis to perform a task that requires a large number of cognitive functions that must be activated under time pressure (Christoffels et al., 2006; Köpke and Nespoulous, 2006). These highly cognitive-demanding experiences are argued to lead to long-term impact in restricted linguistic and cognitive subdomains (Dillinger, 1990; Fabbro et al., 1991; Bajo et al., 2000; Yudes et al., 2013; García, 2014). While several studies report superior WM and concurrent cognitive operations for interpreters (Christoffels et al., 2006; Yudes et al., 2011; Signorelli et al., 2012; Yudes et al., 2013; Babcock and Vallesi, 2017), other studies fail to find an advantage or superior performance among interpreters in several tasks of attention, switching, and inhibitory control. Although we still do not have exhaustive information on interpreters’ cognitive skills, the data that have been discussed have shown that some inconsistencies may be attributed by the lack of controlling for the participants’ linguistic and non-linguistic skills. Those skills vary in degree depending on the task at hand. For instance, different levels of bilingualism will result in different sets of data, generating conclusions that, if not carefully analyzed, can be skewed. Responses to qualitative data (e.g., written survey questions), commonly used to measure perception, must be carefully scrutinized to examine their validity. Some studies that were discussed here, especially those from the 2000s, have presented inconclusive results likely due to the various approaches to comparing groups. It is challenging to find well-trained, highly-proficient interpreters with extensive years of experience who would be available to participate in a study, and to recruit a very large cohort for ample data collection is even more difficult. From our point of view, we have noticed inconsistencies across measures, failures to acknowledge problems of convergent validity, replication reliability, and task impurity. These issues must be resolved as research in this area moves forward.

It is important to keep in mind that in the 1960s and certainly by the early 1970s, interpreting researchers moved from “speculative theorizing” (Gile, 1990) to substantive interdisciplinary inquiries. It is perhaps interdisciplinarity that can help to bridge traditional boundaries and address issues that have been explained in several models, albeit untested: the cognitive model of interpretation (Wilcox and Shaffer, 2005); the semiotic model of interpretation (Ingram, 1978); the communication model (Stewart et al., 1998); the sociolinguistic model of interpreting (Cokely, 1992); the pedagogical model (Colonomos, 1992). Having a better description of participants’ profiles in studies with interpreters is a good start, as a way to clarifying to whom the study findings apply. This could possibly improve the generalizability of the findings as well as diminish internal threats to validity.

## Author Contributions

AF, JS, and JF equally contributed to this conceptual analysis. All authors contributed to the article and approved the submitted version.

## Conflict of Interest

The authors declare that the research was conducted in the absence of any commercial or financial relationships that could be construed as a potential conflict of interest.
